# Unlocking Soil Health and Surface Water Quality Management: A Review on Fluorescent Dissolved Organic Matter (fDOM) in Agricultural Systems

**DOI:** 10.1021/acs.jafc.5c13863

**Published:** 2026-01-12

**Authors:** Md Enamul Haque Moni, Michael Hayes

**Affiliations:** † School of Plant, Environmental and Soil Sciences, Louisiana State University Agricultural Center, 219 Madison B. Sturgis Hall, Baton Rouge, Louisiana 70803, United States; ‡ Louisiana Sea Grant, 5779 Louisiana State University, Baton Rouge, Louisiana 70803, United States

**Keywords:** dissolved organic matter, fluoresces, agricultural soils, water quality

## Abstract

Agricultural runoff is a major source of water quality impairments and is prevalent in areas where agricultural operations focus on maintaining global food security. To alleviate downstream impacts, best management practices are used to cultivate food systems and enhance soil nutrient cycling. When runoff events do occur, tracing the impairments often involves complex and costly methods to determine analyte concentrations and forecast mitigation techniques. Fluorescent dissolved organic matter (fDOM) is an innovative approach to understanding parent source materials and carbon signatures from runoff. Fluorescence and absorbance indices can distinguish intensities of the carbon molecular weight, biological activity, and humification that can trace the environmental availability of carbon sources. Comprehensive data sets can be combined using parallel factor analysis (PARAFAC) to determine parent source components. Integrating these analyses can provide real-time high-frequency data to empower policymakers and land managers to make informed decisions aimed at reducing the environmental degradation associated with modern intensive agriculture.

## Graphical abstract

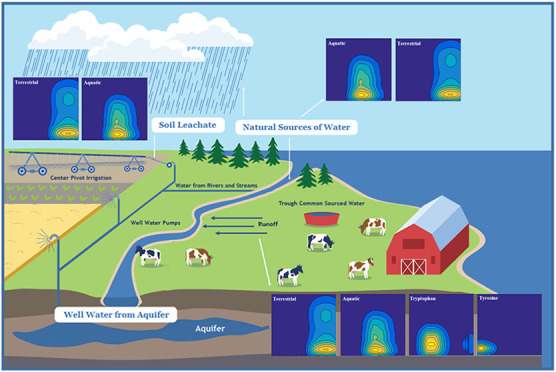

## 1 Introduction

Anthropogenic activities, such as farming, greatly influence the type of land use, runoff dynamics, and fate of environmental impairments. Intensified modern forms of agriculture are imperative for global food security but have led to serious environmental impact and degradation. For example, agricultural output has increased 400% in the last 60 years globally, and, at the same time, the global population increased 260%, which ultimately led to a 53% increase in per capita agricultural output.^1^ Nonpoint source pollution generated from agricultural runoff is a well-documented and major concern that creates a significant threat to the nearby aquatic ecosystem.^2–4^ Agricultural runoff can be a complex combination of pollutants, including pesticides, fertilizers, and organic matter, which immediate impact on downstream rivers and coastal areas.^2^ Specifically, nutrients like nitrogen (N) and phosphorus (P) are carried into the aquatic system and trigger eutrophication, resulting in algal blooms and oxygen depletion.^5^ On a global scale, agriculture is the largest contributor to water quality degradation. It is estimated that every year globally, 2250 km^3^ of effluent is discharged into the environment, and 1260 km^3^ of that comes from agriculture alone. According to the United States National Assessment, agricultural runoff is the leading cause of water quality impairment to rivers and streams, the second largest source of impairments to wetlands, and the third leading source for lakes.^6^ Each year in the United States, approximately half a million tons of pesticides, 12 million tons of N fertilizer, and 4 million tons of P fertilizer are used to support agricultural production.^2^ Though agricultural runoff impairments are often associated with fertilizer nutrients, organic matter can provide insight into many different sources of environmental impact, including turbidity and sedimentation from land loss, low dissolved oxygen through microbial and plant breakdown, and intensity of manure-based runoff from livestock operations, and can be correlated to areas of high nutrients in watersheds. With the continued use of anthropogenic compounds and volume runoff draining from agricultural land, it is crucial for scientists to better understand the transport and fate of organic matter that originates from agricultural fields to maintain environmental quality and sustainable land management.

Dissolved organic matter (DOM) is the most reactive and mobile fraction of the organic matter in natural systems.^3^ It plays a crucial role as a vector for nutrient and contaminant transport while influencing the ultimate fate of those nutrients and contaminants.^7,8^ DOM plays a significant role as a combination of a heterogeneous mixture of organic compounds.^9^ In addition, it is a major part of the global carbon cycle, an energy source for heterotrophic microorganisms, and an important factor of surface water in terms of photochemical and biological processes.^10^ The chemical characterization of DOM derived from agricultural runoff has a direct influence from the agricultural practices like fertilizer application, tillage operation, and agricultural waste management.^11^ Therefore, to understand the biogeochemical cycles of carbon, nitrogen, and phosphorus in the agricultural landscape, the characterization of DOM can be an innovative tool to trace carbon through environmental systems.

Natural DOM is a very complex collection of organic matter that is soluble and rich in organic carbon (C), nitrogen (N), and phosphorus (P).^12,13^ Found in all natural aquatic systems, DOM is defined as the portion of organic matter that can pass through 0.2 to 0.7 μm filters (most commonly 0.45 μm filter for research).^14,15^ Filtration processes are used to separate DOM from particulate organic matter (POM), as these species are easily transformed through chemical and physical processes within the water column.^16^ A portion of that DOM is optically active by absorbing and emitting light in the ultraviolet (UV) and visible spectral region. Chromophoric and fluorophoric signals are caused by optically active DOM functional groups. These signals can vary based on the molecular compounds and functionality of diverse parent source materials with different biogeochemical origins.^17^ The portion of DOM that absorbs light is called chromophoric or “colored” DOM (CDOM), while the fluorophoric subfraction known as fDOM re-emits the absorbed light.^18^ Identifying the CDOM and fDOM group specifically is a challenge; however, previous research suggests that the aromatic, phenolic, and carbonyl functional groups are correlated to CDOM, and fluorescent amino acid, polyphenol, and organic acid are correlated to fDOM signals within various environmental systems.^18^ Based on the origin, the DOM pool is made of either autochthonous or allochthonous organic molecules.^19^ In a broader sense, autochthonous materials are derived from within the aquatic system, like aquatic macrophytes, phytoplankton, and sediments, whereas allochthonous materials come from outside of the system, essentially degraded terrestrial materials.^20,21^ In some cases, dissolved organic carbon (DOC) is used interchangeably with DOM due to the C constituting greater than 50% of the weight of natural organic matter in various systems.^5^ In addition to this, the fDOM intensity is strongly related to the concentration of DOC and can be used to determine a specific UV absorbance (SUVA) ratio for concentrations of the different molecular weight carbon species.^22,23^ Studies including Del Cid, 2019 and Fernández-Pascual et al., 2023 have used fDOM as a proxy for DOC analysis in drinking water and disinfectant byproducts as well.^22,24^ Due to the lack of literature that uses fDOM measurements in agriculture and the high abundance of C in agricultural soil systems, this review will rely on studies that focus on both DOM and DOC in agricultural systems, thus using the terminology interchangeably.

Methods to determine fDOM can be a powerful and reliable optical tracer for characterizing DOC. However, fDOM provides structural and source-specific results by using the intrinsic fluorescence of aromatic moieties and protein-like substances, instead of bulk DOC measurement. Over the past three decades, numerous techniques have been developed to interpret excitation–emission matrices (EEMs) of natural waters, ranging from simple peak-picking approaches to advanced statistical models. A notable intrinsic property of DOM is the specific wavelength of light absorption and re-emission, which allows scientists to use these values as a natural tracer. Organic matter sources such as manure, microbial byproducts, or leachates produce unique fluorescent “fingerprints”.^5^ In addition, fluorescence spectroscopy has proven to be a rapid, sensitive, and nondestructive technique for characterizing DOM. In particular, the EEMs provide a comprehensive three-dimensional map of the fluorescent landscape for each sample.^10^ Although these data sets are complex, there have been major advancements in indicator analysis by multivariate statistical tools, most commonly PARAFACs. Unlike traditional peak-picking methods, PARAFAC enables a more robust and quantitative characterization of DOM by mathematically decoding the overlapping signals in EEM data sets.^9,25^

There are several ways in which the EEM data set could be analyzed for the characterization and measurement of fDOM components. Chow et al., 2022 mentioned four major techniques, as they are used by different scientists at different times.^15^ For example, fluorescence peaks have been used as a technique to determine discrete fDOM components (protein-like component vs humic-like components, etc.).^26^ Later, these qualitative insights were complemented by quantitative indices like fluorescence index (FI), biological index/freshness index (BIX), and humification index (HIX), which provide sample-specific information to better understand carbon impact on environmental systems.^27,28^ Computational development enables more integrative techniques like fluorescence regional integration (FRI) that operationally partitions the EEM among five regions, such as tyrosine-like, tryptophan-like, fulvic acid-like, soluble microbial byproduct-like, and humic acid-like.^29^ At each region, the volume is numerically integrated and normalized, resulting in an estimated relative contribution with accuracy increased by a smaller increment in wavelength and composite Simpson’s rule.^30^ On the other hand, PARAFAC analysis is more effective and has revolutionized fDOM characterization by extracting complex fluorescence EEMs into independent components mathematically.^31^

Fluorescence spectroscopy coupled with PARACAC analysis has been widely applied to study marine environments and wastewater management systems;^9,25^ however, there is insufficient literature to define its use in the agricultural system. A comprehensive study of the agricultural system is necessary to optimize agricultural management strategies and build a strong base for future research. This review will find out the gap and deliver a critical overview of the use of fluorescence spectroscopy of DOM in agricultural runoff, while exploring the relationship between the characteristics of fDOM to understand transport and fate of parent source carbon in agricultural systems.^3,5^ The paper will cover the fundamentals of fluorescence spectroscopy techniques, sources and dynamics of fDOM in agricultural landscapes, the application of fDOM in nutrient prediction, optimization of management practices, and current and future challenges in research.

## 2 Fundamentals of Fluorescence Spectroscopy

### 2.1 Principles of Fluorescence Spectroscopy

Aromaticity is an intrinsic property of optically sensitive DOM, and fluorescence spectroscopy is a very fine-tuned scientific technique based on the photophysical process.^18^ In this process, the fluorophore absorbs light energy, resulting in an electron jumping to an excited higher energy state. Later on, these molecules lose energy and return to their initial state through vibration and by emitting energy as a photon of light.^25,32^ The wavelength of the emitted light is longer (lower energy) than that of the excitation light. The analysis of the fluorescence data was done by using the excitation–emission matrix (EEM), which is a three-dimensional plot that can map the intensity across a range of excitation and emission (Ex/Em) wavelengths at the same time. EEM delivers a comprehensive fluorescent fingerprint for every sample by scanning a series of excitation wavelengths and capturing the full emission spectrum at each step.^33,34^ Distinct peaks and regions of high intensity from various fluorophores within the DOM pool enable EEM for quantitative and qualitative characterization of fDOM.^5^

### 2.2 Data Analysis for Indicators

From the initial data sets, calculating specific indicators can provide impactful information from within EEM. This is essential to understanding the underlying characteristics of the fDOM compounds. There are several fluorescence indices that have been used to assess the fDOM characteristics quickly from a specific wavelength EEM point. The fluorescence index (FI) distinguishes between terrestrial (allochthonous) and microbial (autochthonous) sources. It allows for differentiation between the terrestrial (FI value lower than 1.4) and microbial (FI higher than 1.9) sources of DOM.^3,35^ FI > 1.8 suggests microbial sources (e.g., manure, wastewater, algal production), and FI < 1.4 suggests terrestrial sources (e.g., soil, plant litter).

$$fluorescence\;index\;\left(FI\right)=\frac{450\;nm\;emission}{500\;nm\;emission}\;at\;370\;nm\;excitation$$

The biological index (BIX) indicates the freshness of the DOM. If the BIX value is greater than 1, it indicates newly produced DOM from biological or microbial origin. On the other hand, a value less than 0.6 means a little fresh material.^36,37^ Higher values (>0.8) suggest a greater contribution from autochthonous biological activity.

$$bio\log ical\;index\;\left(BIX\right)=\frac{380\;nm\;emission}{430\;nm\;emission}\;at\;310\;nm\;excitation$$

The humification index (HIX) identifies decomposed, terrestrial organic matter that has a higher HIX value, which also represents a higher degree of humification and aromaticity.^38,39^ Higher values suggest more humified, complex, and aromatic materials of a terrestrial origin. Lower values indicate freshly produced and less humified DOM.

$$\mathrm{humification}\;\mathrm{index}\;\left(\mathrm{HIX}\right)=\frac{\left[\sum_{\mathrm{em}}\left(435-480\;\mathrm{nm}\right)\right]}{[\sum_{\mathrm{em}}\left(300-345\;\mathrm{nm}\right)]+[\sum_{\mathrm{em}}\left(435-480\;\mathrm{nm}\right)]}\;\mathrm{at}\;254\;\mathrm{nm}\;\mathrm{excitation}$$

Specific ultraviolet absorption at 254 nm (SUVA_254_) has been widely used as a proxy for DOM aromaticity. Typical values range from 0.6 to 5.3 L mg C^–1^ m^–1^ in natural waters, expressing 5–40% aromatic carbon,^40^ although higher values have been observed in extracted humic and fulvic acids.^41^ SUVA_254_ provides valuable insights into DOM composition, sources, and reactivity in fDOM research.

$$\mathrm{specific}\;\mathrm{ultraviolet}\;\mathrm{absorbance}\;\mathrm{at}\;254\;\mathrm{nm}\;\left({\mathrm{SUVA}}_{254}\right)=\frac{A_{254}}{\mathrm{TOC}\left(\frac{\mathrm{mg}}{L}\right)}$$

### 2.3 PARAFACs Analysis

This multivariate analysis method is standard for disseminating a series of EEM data sets into separate underlying fluorescent components.^34^ This differs from the simple indices, as PARAFAC can model the entire EEM landscape to produce parent source components (Figure 1). It can mathematically differentiate the overlapping fluorescent signals and convert them into a spectrally independent component. Individual components are identified with their specific excitation and emission spectra in addition to their relative concentration.^9^ This statistical technique allows scientists to detect and quantify fDOM components, such as humic-like and protein-like materials, from a complex DOM pool. Additionally, the components can be compared via research platforms such as OpenChrom to determine the correlations and consistency of modeled components in various types of land use and natural events. The component analysis ranges from monitoring weather events, wastewater treatment processing, and wetland carbon sequestration.^42–44^ This also helps build a significant relationship between fDOM and water quality parameters like nutrient concentration or bioavailability.^45^

**Figure 1:**
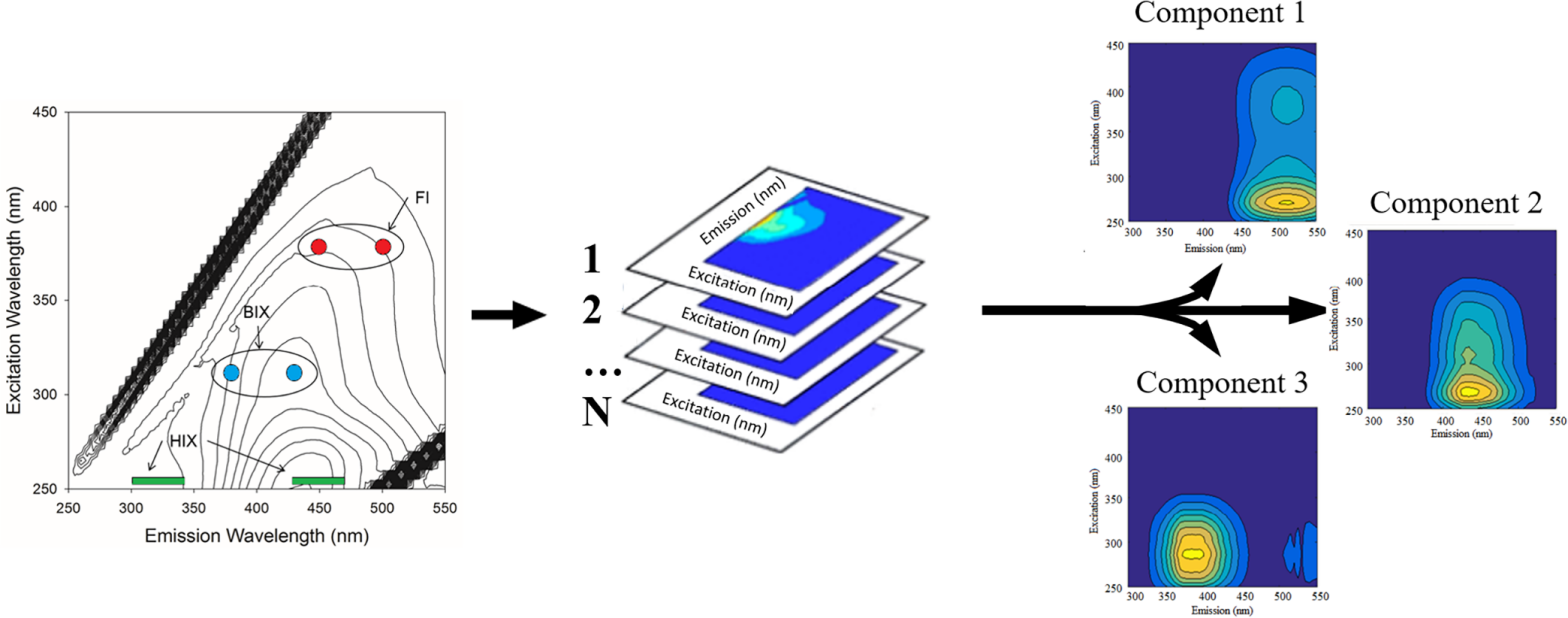
Process of producing the components using PARAFACs. The single EEMs (left) are used to determine the indicators from the formulas given in Section 2.2. These individual EEMs are compiled in full data sets (middle) of an environmental system and processed using the PARAFACs statistical analysis to determine components (right) in order of highest contribution.

### 2.4 Components of fDOM

While only a part of the DOM re-emits light, fDOM is crucially informative and serves as a powerful tool to characterize and identify the source of the broader DOM pool.^10,32^ There are many complex chemical characterizations that fDOM can visually separate using PARAFACs, but it is broadly categorized into two main groups: humic and protein-like substances. Humic substances are complex and higher-molecular-weight aromatic compounds that originate from the decomposition of plant and microbial matter. They are very common fDOM components in soil-influenced and terrestrial systems. Humic substances are subdivided based on their solubility in various pH levels, for example, fulvic acids (soluble at all pH levels) and humic acid (insoluble at acidic pH).^32^ Humic-like fluorescence is typically associated with allochthonous (terrestrial) inputs such as agricultural runoff into aquatic systems. This component is observed at a longer emission wavelength (greater than 400 nm).^45,46^ Humic-like substances are also characterized by peak A, peak C, and peak M, where peaks A and C are considered as terrestrial sources and M as marine.^26^ Protein-like substances are primarily tryptophan and tyrosine (originating from aromatic amino acids), free or bound within peptides and proteins. The fluorescence of protein-like substances occurs from shorter wavelengths (less than 380 nm).^32,47^ These substances indicate autochthonous (aquatic) or microbial sources presenting the latest biological activities (e.g., from algae, bacteria) or fresh organic matter decomposition (e.g., manure, crop residue).^48,36^ Peak T (tryptophan-like) and peak B (tyrosine-like) are named after the amino acids that they resemble and can indicate microbial processing in an aquatic system. Table 1 shows common components with the corresponding Ex/Em range and a brief description of the source. The relative availability of these compounds in the agricultural system provides critical information about the origin and fate of DOM within the watershed. By applying these indices and statistical analytical tools, complex fluorescence signatures from agricultural systems can be understood and transformed into perceivable information on the DOM sources, characteristics, transport, and fate in aquatic systems.

**Table 1:** Descriptions of Components with Ex/Em Ranges

**component**	**excitation/emission (Ex/Em) maxima (nm)**	**source and characteristics in agricultural landscapes**	**key references**
humic-like (peak C)	Ex: ∼320–360/Em: ∼420–460	terrestrial, allochthonous in origin, peak C is derived from the decomposition of higher plant materials (e.g., crop residues, soil organic matter) and is typically associated with higher-molecular-weight, aromatic, and more recalcitrant organic matter	^26^ and^46^
humic-like (peak A)	Ex: <260/Em: 448–480	terrestrial in origin, peak A is often associated with small humic-like or fulvic acids and is often found in soil leachates and surface runoff in agricultural environments and wetlands in relationship with more easily degraded compounds	^26^ and^46^
humic-like (peak M)	Ex: ∼290–320/Em: ∼370–430	terrestrial, microbial in origin, peak M is often associated with marine or microbially derived humic-like substances and is associated with soil organic matter breakdown in water systems	^26^ and^46^
tryptophan-like (peak T)	Ex: ∼270–280/Em: ∼330–368	microbial/autochthonous origin. a strong indicator of fresh, labile, proteinaceous materials. associated with microbial activity, wastewater, and animal manure. serves as a key tracer for fecal contamination	^26^ and^32^
tyrosine-like (peak B)	Ex: ∼270–275/Em: ∼300–310	microbial/autochthonous origin. derived from the amino acid tyrosine. Indicates biological production and microbial byproducts, often found in conjunction with tryptophan-like fluorescence, but can be less intense	^26^ and^32^

## 3 Sources of fDOM in Agricultural Systems

Modern farming is associated with natural environmental elements and diverse inputs, which constitute the fDOM pool found in agricultural systems. Understanding the sources and fates of organic matter in agriculture would be a key factor in mitigating impacts on the environment, especially in downstream water systems. The formation of fDOM in any terrestrial ecosystem comes from the decomposition of natural soil organic matter (SOM) and plant residues (Figure 2). This process creates the natural composition of soil by releasing high-molecular-weight, aromatic carbons, which form humic-like substances.^49^ Agricultural soils are heavily supplemented with human inputs. These anthropogenic inputs increased the amount of organic matter and impacted degradation processes.^2,4^ Table 2 summarizes common sources of fDOM that come from the agricultural management system.

**Figure 2:**
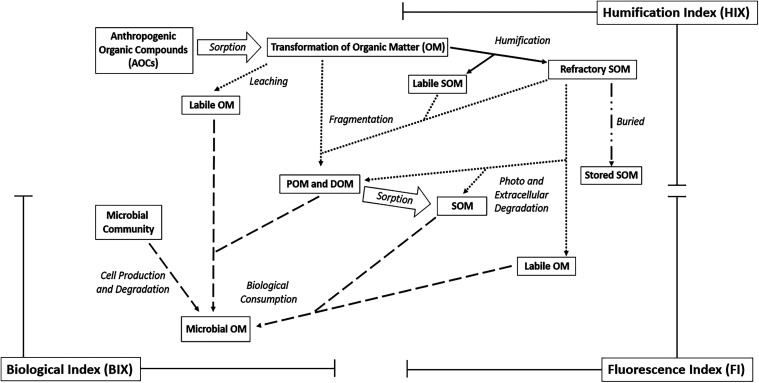
Illustrates various pathways for the fate and transport of organic matter through an environmental system. These pathways create unique parent source materials that can be traced through the fDOM analysis.

**Table 2:** Sources of Agriculture Management and Potential Impact on fDOM

**source**	**description for agricultural systems**	**key references**
crop residue	crop residues derived from the postharvest management of field crops like sugar cane, wheat, or rapeseed straw are significant sources of agricultural fDOM. the residue releases high-molecular-weight, terrestrial organic matter during field decomposition. as farming operations progress, organic matter starts decomposing on the surface and subsoil. this releases humic-like and protein-like substances with a distinct fDOM signature, which can be transported through the system during rain events	^50^ and^51^
fertilizer application	inorganic mineral fertilizers might not directly contribute to the fDOM pool, but they have a profound impact on its composition by influencing soil microbial activity. these fertilizers can increase microbial activities, which in turn accelerate the decomposition of the SOM. this catalyst can reduce the leached DOC and transform the DOM composition into a more microbial-like signature. on the other hand, organic fertilizers, for example, pig slurry, directly supply labile DOM and nutrients to further enhance microbial activities and modify the fDOM signature of the soil leachate	^52^ and^53^
soil amendments	soil amendments also alter and contribute a unique fDOM signature. for example, biochar leaches a specific DOM characterized by a humic-like, aromatic structure. this is due to the unique method of creating biochar, which sequesters carbon, leading to increased HIX values. conversely, composts can release more stable fulvic and humic-like substances, with traces of recent microbial processing from the nutrient conversion process	^54^ and^55^
manure and animal waste	the application of manures and compost as organic fertilizers in the crop field, as well as livestock and poultry operation effluents, produces a large amount of protein-like, microbial-derived organic matter. these organic matters are high in tryptophan- and tyrosine-like fluorophores that reflect the disintegration of proteins and their origin from the metabolism of microbes. additionally, the parent source material can be used to identify beneficial uses of agricultural livestock wastewater by comparing its impactful carbon signature to the existing systems	^36^,^55^, and^56^

The diverse fDOM produced by various forms of agriculture creates a unique opportunity for the innovative use of fluorescence spectroscopy coupled with PARAFACs analysis to fingerprint DOM for a better understanding of agricultural systems. Table 3 shows how this could be applied to humic-like substances (e.g., fulvic- and humic-acid-like components) from decomposed terrestrial organic matter post harvest or impacts of best management practices (BMPs) such as burning crop residuals. For these cases, the signature would be complex aromatic fluorophores, with vital information on the richness of plant materials as a source of nutrients for future crops through their decomposition.^55,57^ Additionally, signatures like tryptophan- and tyrosine-like components are strong indicators of recent anthropogenic or biological inputs. They are credible tracers for pollution from animal agriculture, where livestock effluents and animal manure are major sources.^36,56^ Statistical methods like PARAFACs are often used to locate tryptophan and tyrosine components from the leachates of swine manure and poultry litter.^50,55,56^ By sampling soil porewater or surrounding watersheds, we can help scientists trace the origin of fDOM to implement or validate BMPs. Whether identifying a higher protein-like proportion to indicate pollution from livestock waste or using the dominant proportion of humic-like components to identify soil erosion, the EEM-PARAFAC tool will provide the capacity to bridge fluorescent signatures to agricultural land use and management. The association of organic matter as an environmental pollutant is often overshadowed by other sources’ magnitude, like the transport, fate, and microbial processing of nitrogen and phosphorus, which serves as the central driver of the overall environmental impact of nonpoint source pollution.^14^

**Table 3:** Sources and Components of fDOM in Various Agricultural Systems

agricultural system	dominant fDOM signature (source)	key PARAFAC components/peaks and wavelengths (Ex/Em nm)	characteristic index ranges	source
intensive livestock production (LEs)	protein-like (∼68% total fDOM). autochthonous/microbial origin	tryptophan-like (peak T/C1): 272/354. tyrosine-like (peak B/C2): 270–280/340–360 (tyrosine observed in slurries)	FI: high range, typically >1.9. BIX: high, >0.8 in 95% of samples. HIX: relatively low (<10 is common)	^5^ and^36^
dryland farming/general agricultural effluents (AEs)	primarily humic-like (∼64% total fDOM). terrestrial and autochthonous sources	terrestrial humic-like (peak C/C4): consistent with 250(330)/430 or 255(360)/455 nm. fulvic-like (peak D/C3): similar to soil-enriched materials. UVA/UVC humic-like	FI: lower range, typically 1.04–1.52. AEs soil FI: 1.08–1.31. BIX: high, all samples >0.8. HIX: higher than LEs, typically 1.5–9.0	^11^ and^36^
paddy fields/organically amended soils (varies with depth)	mixed terrestrial/microbial source depending on depth; affected by straw/residue inputs	humic acid-like (C1): 255/450 nm. fulvic acid-like (C5): 220(290)/420 nm. tryptophan-like (C4, C6): 230(275)/340 nm and 220(270)/290 nm. solubility-like microbial metabolite (C3): 200(255)/340 nm	FI: decreases with depth, reflecting a shift from terrestrial topsoil to microbial deep soil influence. BIX: increases with organic amendment and depth, indicating new microbial DOM generation. HIX: higher in topsoil with straw amendment (more humified), indicating decay	^50^
compost/soil organic amendment (maturity assessment)	humic-like substances evolve from protein-like substances as composting progresses	humic-like (C1): 2,30,330/410 nm. fulvic-like (C2): 2,50,350/450 nm. protein-like (C3): 2,20,280/340 nm	compost is assessed as mature when log(scores) of C1 and C3 are higher than 3.69 ± 0.06 and 3.49 ± 0.09, respectively. no correlation found between PARAFAC scores and C/N ratio, TOC/TN ratio, or maturity index (MI)	^55^

## 4 Factors Impacting the Dynamics of Agricultural Runoff

There are numerous agricultural management and environmental factors for which the fDOM fingerprinting is dependent. The dynamics of fDOM are very complex, and the interaction of human management at the farm level, overall environmental, and hydrological drivers influences these dynamics at the watershed level.^45,52^ Synthesizing all the variables into component models can be a powerful tracer to identify major concerns in agricultural management and drive more adaptive practices to mitigate water impairments.^36,57^ The optical properties of fDOM compounds offer sensitivity to accurately characterize the changing pathways and sources of DOM fate in various environmental systems as seen in Steinmuller et al. (2020) where PARAFACs were used to study SOM properties in erosion events.^58^ To facilitate a better understanding of various impacts on agricultural DOM, human management practices at the farm level and external factors like environmental and hydrology are thoroughly outlined in the discussion below.

### 4.1 Farm Management Practices: The Anthropogenic Imprint on fDOM

Among the anthropogenic activities, agricultural management practices (Table 4) are one of the primary responsible factors for the modification of carbon and nutrient cycles in the environment. Management decisions such as tillage, fertilization, irrigation, cover cropping, and livestock management directly influence the quantity and quality of DOM available for export, creating distinct fluorescent fingerprints that can be traced into adjacent aquatic ecosystems.

**Table 4:** Summary of Farm Management Practice Effects on fDOM in Runoff

**management practice**	**effect on DOM concentration**	**effect on humic-like fDOM**	**effect on protein-like fDOM**	**typical impact on fluorescence indices**	**primary mechanism and key references**
conventional tillage	increased erosion may elevate total OM; WEOM concentration can be higher than NT	dominant component in leachate from the homogenized soil profile	lower relative to the NT surface runoff	HIX may increase due to soil processing	soil homogenization, accelerated SOM decomposition, and erosion^49^
no-till	pulsed increase during storms (“first flush”); may decrease baseflow concentration	lower relative abundance in the first flush runoff	high concentration in the first flush from surface residues	FI and BIX increase during first flush events	surface residue accumulation, advective transport during overland flow^49^
inorganic fertilizer	increases soil DOM concentration	relative decrease as microbial components increase	strong increase in microbially derived components	FI and BIX increase	stimulation of microbial processing of native SOM (“priming effect”)^52^
organic fertilizer (manure)	strong increase, especially in labile fractions	present, but masked by an intense protein signal	very strong increase; dominant fDOM fraction	FI and BIX increase significantly	direct input of labile, protein-rich organic matter and microbial byproducts^36^
cover cropping	can increase DOC in leachate due to residue decomposition	increased terrestrial input from decomposing biomass	increased microbial/labile input from decomposing biomass	BIX and FI may increase post termination	reduces erosion but adds a large pool of fresh organic matter for decomposition^48^
livestock grazing	significant increase in runoff from pastures/feedlots	low relative to the protein-like signal	very strong increase: signature used as a fecal tracer	FI and BIX are very high	direct excretion of manure and urine onto the landscape^36^

#### 4.1.1 Tillage Operation

Tillage is a prominent agricultural management practice by which crop fields are physically altered by farmers. This makes changes to the structure of the soil, organic matter distribution, and hydrological pathways, thus altering the DOM dynamics. Soil cultivation creates aerobic conditions in the soil and makes oxygen available, resulting in enhanced microbial activity and the decomposition of soil organic matter.^59,60^ This process enriches the soil substrate with DOC, which is more sensitive to tillage operations compared with the total organic carbon pool in the soil. This means that soil without any vegetation loses a significant amount of carbon as DOC.^49^ Conventional tillage increases the mobility of soluble organic molecules by accelerating microbial breakdown, while the reduced tillage technique possesses a minimum soil physical disturbance and keeps the labile carbon in the upper soil layers. Haynes (2000) showed that the addition of grazed grassland to a cropping system holds a greater amount of labile organic carbon, and under such conditions, greater stability of DOM is found.^61,62^ The quantity and stability of DOM are influenced by the interaction between tillage and land with moisture, soil texture, and biological activity. For example, the transformation of pasture to arable land usually decreases DOM content; at the same time, perennial cover or reduced disturbance could reduce carbon loss and promote DOM retention.^62^ These results show the importance of choosing a tillage strategy to maintain DOM stocks in agricultural soil.

#### 4.1.2 Fertilizer Application

Organic fertilizers like animal manure and compost directly contribute allochthonous fDOM in the agricultural soil.^49^ Manure and slurries are composed of high-labile, protein-like organic matter with tryptophan- and tyrosine-like components. These amino acids serve as a reliable indicator of manure-related pollution in aquatic systems.^36^ Other non-animal waste compost materials can supply more processed materials in the organic matter pool (e.g., aromatic-like).^55^ Inorganic fertilizers have a significant influence on soil microbial activity. For example, the input of readily available nitrogen reduces nutrition limitation, inducing a priming effect, which speeds up native SOM decomposition and crop residue.^52^ This enhanced microbial metabolism can shift DOM composition toward smaller molecular size and fresher, microbially influenced fDOM components. The resulting leachate signature in these cases is rich in more labile, microbially influenced components, showing that the bioavailability of exported DOM can significantly vary based on soil texture, fertilizer type, and time since application.^52^

#### 4.1.3 Irrigation Practices and Cover Cropping

Microbial metabolism and decomposition rate increase with the effect of irrigation, and thereby, the production of autochthonous microbial fDOM components also increases. The principal consequence of irrigation is the increase in soil moisture, which alleviates water scarcity for soil microbes and improves metabolism, decomposition rates, and overall production of microbial fDOM components.^63^ In addition, irrigation acts as the primary transport vector for the DOM to move through the stratification of soil layers. Mechanical watering also increases the leaching process of DOM through the soil profile and export by subsurface drainage or surface return flows.^3,63^ The chemical structure of DOM can also be affected by extreme wet or dry conditions of the soil. For example, wet soil conditions are associated with the export of more aromatic and complex DOM, whereas the period of dryness is linked to diminishing the structural complexity and photodegradation of surface soils.^64^ In the case of fallow fields, to help mitigate the effects of water transport, cover crops are a common BMP that adds organic carbon to improve soil health and protect water quality by reducing leaching.^65^ However, some studies showed that there are no changes in bulk soil organic carbon as a result of cover crops in a short growing window, rather some positive changes in terms of water-extractable organic matter.^66^

#### 4.1.4 Livestock Management

In some watersheds where animal agriculture is significant, livestock play the leading role in fDOM input. Manure and urine are mainly mobilized by runoff and expose a complex source of nutrients with microbially derived DOM in a nearby aquatic system. A distinct type of fDOM signature is generated from livestock agriculture, particularly tryptophan-like fluorophores, and characterized by intense fluorescence in the protein-like region of the EEM spectrum.^36^ Specification of the distinctive spectral fingerprint is very dependable and allows the PARAFAC model to differentiate between the fDOM signatures from different animal sources, like poultry.^55^ The EEM coupled with PARAFAC can be used to trace the flow of livestock effluents and impairments in environmental systems to promote more targeted mitigation techniques.

### 4.2 Environmental and Hydrological Factors

While farm management practices dictate the source and initial characteristics of the fDOM pool, the ultimate expression of fDOM in runoff is modulated by various environmental and hydrological factors.

#### 4.2.1 Rainfall Intensity and Duration

Agricultural practices can alter DOM in surface water either by adding additional anthropogenic compounds or by directly contaminating DOM through runoff. In both cases, the variability in DOM concentrations can be affected by many environmental factors like temperature, precipitation, and dissolved oxygen.^67,68^ Seasonal variability also affects the type of DOM transported to surface water; for example, precipitation can increase allochthonous DOC inputs through streams and overland flow. The frequency and volume of precipitation can change the residence time of the DOM in the water body.^69^ The events of precipitation are the primary driving forces of fDOM export in the agricultural system. High-intensity rainfall often creates overland flow and leads to a “first flush” effect where the initial runoff is rich in labile, protein-like components that leached from crop residues and manure.^45^ On the contrary, lower intensity of rainfall with longer duration enhances infiltration, flushing more processed, aromatic, humic-like fDOM from the soil matrix.^45^

#### 4.2.2 Soil Type and Topography and Land Use

Soil properties, such as texture and mineralogy, directly impact the fate of DOM through adsorption. For example, smaller components such as protein-like compounds are mobile in the soil solution, whereas clay minerals and metal oxides have a strong affinity for large, aromatic, humic-like fDOM components and make them less mobile.^49^ Soil acts as a natural chromatographic system with this selective retention capacity and fractionates various forms of fDOM as it percolates downward into the soil. As a result, water that reaches a stream through subsurface pathways could be particularly depleted of terrestrial, humic signature and comparatively rich in protein-like compounds.^52^

The flow path and water residence time are greatly affected by topography, where a steep slope allows rapid surface runoff and a flatter surface promotes infiltration and leaching of water, thus maintaining DOM.^70^ At any given watershed outlet, the fDOM signature is a complex and integrated signal reflecting various land uses. To resolve this complexity, PARAFAC modeling acts effectively in deconstructing the mixed signals and identifying parent source components through Ex/Em values.^57^ The types of fDOM found in environmental systems can vary based on the use of the land and the type of soil substrate. For example, heterogeneous signature that includes both terrestrial (crop residue) and microbial (fertilizer and manure) sources are derived from agricultural systems, while allochthonous, terrestrial humic-like fDOM are found in forest lands.^50,71^ In the case of city or municipality areas, distinct anthropogenic kinds of fDOM sources, such as wastewater treatment plant effluents, are found, which are very rich in protein-like compounds.^57^

#### 4.2.3 Seasonality and Temperature

The concentration and kind of DOM can vary depending on the variability in seasonal precipitation, air and water temperature, and solar radiation. For agricultural runoff, the seasonal pattern has a great impact on fDOM, guided by the cycles of temperature, hydrology, and biological activity.^43^ In the growing season, there is an increase in freshly produced labile fDOM that comes from crop root exudates and enhanced microbial metabolism. This process results in a signature rich in protein-like and microbial humic-like components and increases BIX and FI values.^36^ During the cool season in fall and winter, the decomposition of crop residues can flush more aromatic, humic-like compounds into the streams, resulting in higher HIX and SUVA_254_ values.^56^ As the temperature has a critical control on fluorescence (fluorescence intensity increased as temperature decreased), the effect of temperature must be taken into consideration and corrected when conducting field studies.^72^ Photodegradation can decrease the concentration of DOM by mineralizing DOC on a daily basis.^73,74^ Watras et al. (2016) showed that fDOM concentrations reach their highest at night and decrease during the day in multiple Wisconsin lakes.^75,76^ Additionally, seasonal changes in precipitation allow allochthonous carbon input through runoff,^69^ and changes in temperature and dissolved oxygen can enhance DOM mineralization.^77,78^ Dinsmore et al. (2013) showed temperatures to have a positive correlation with DOC in streams because of increasing biological production from allochthonous sources in a broad scenario.^79^ This trend can also be applied to air temperature, which has proven to be a factor that stimulates the soil enzyme activity and increases the input of soil-derived DOC to the aquatic system.^80^

## 5 Applications of fDOM in Agricultural Monitoring and Management

The understanding of the source of origin of fDOM and the knowledge about the factors that control fDOM dynamics enable us to transfer the foundational idea into practical applications. Several fluorescence indices provide impactful information about the source and nature of organic matter in an environmental system. However, when they are coupled with PARAFAC, the data become a comprehensive and multifaceted tool for fingerprinting DOM impairments. This can provide insight for practical BMP applications, such as in the fields of wastewater treatment and water resource management. The high sensitivity and source-specific nature make this process very useful for the application of fDOM analysis in the context of an agricultural system.

### 5.1 Source Tracking and Apportionment

The ability to fingerprint DOM in water and trace the source of impairments could be a major application of fDOM analysis. Agricultural runoff is often mixed with different kinds of anthropogenic land use, for example, urban stormwater and industrial effluents, so tracking the original source is a crucial task for the authority to manage the waste and impairments properly. The EEM coupled with PARAFAC has proven to be an exceptionally effective tool to solve this kind of real-life challenge.^57^ Previous research showed that different kinds of pollutants left distinct fluorescent signatures on the receiving water. For example, the use of PARAFAC models can decode complex EEMs and isolate humic-like terrestrial origin components that have come from runoff and protein-like components that have commonly come from anthropogenic waste.^57,81^ On the other hand, tryptophan-like fluorescence is very often identified as a unique PARAFAC component for tracing domestic sewage and animal manure because of the high concentration within these kinds of waste.^36^ With the comparison of the fDOM composition with a known or standard fingerprint of probable sources, scientists can use several other models (e.g., principal component analysis, PCA) to quantitatively measure the ratio of the pollution load.^81^ This method has been successfully used in Taihu Lake Basin in identifying agricultural activities and home sewage, with a proportion contributing 42 and 21%, respectively, as a dominant pollutant source.^81^ This enables the managers and the responsible authority to take measures against impairments by effectively making a distinction between the parent source material.

### 5.2 Tracing and Predicting Nutrient (N and P) and Contaminant Fate

The use of the fDOM technique is not only limited to identifying and tracking the DOM sources, but it has also been increasingly used as a proxy to track the changes and transport of several other key agricultural components (e.g., N, P, etc.). The direct measurement of nutrient concentration could be labor-intensive and costly, whereas a strong correlation between specific fDOM components and different forms of nutrients would offer a cost-effective and promising alternative. For example, total nitrogen and total phosphorus are found to be strongly correlated with protein-like fluorescence, while distinct humic-like components have a strong relationship with nitrate (NO_3_^–^).^5,81^ In Qi et al. (2023), both agricultural and livestock effluents were identified as terrestrial humic-like components (peak D) and classified as a dependable proxy for total phosphorus.^36^ Biological oxygen demand, which is an indicator of bioavailable organic pollution, was predicted by tyrosine-like components (peak B).^36^ This relationship is dependent on the cotransport of a specific fDOM fraction with nutrients in the same hydrological condition (e.g., N, P, and protein-like DOM from manure), and the DOM itself acts as a direct modulator of nutrients.^49^ The potential use of fDOM will be a great advancement as a real-time proxy in pollutant source tracking and nutrient monitoring in the agricultural system.

### 5.3 Assessing the Best Management Practices (BMPs) and Real-Time Monitoring

In agriculture, BMPs are essential for maintaining profitability and mitigating adverse environmental outcomes. The fDOM analysis provides a sensitive and nuanced tool for this assessment of the BMPs and enables us to detect subtle changes in water quality that might not be apparent from bulk measurements alone. For instance, in a successful wetland system, it is expected to have a reduction in the intensity of labile and protein-like components and a change toward more processed humic-like material, proving the microbial processing and settling of organic matter within the wetland.^57^ As fDOM analysis is quick, it can trace the fate of specific sources. This can provide information on the downstream impact from processing and can be used as a rapid validation of the BMP performance. Despite the advances in science, there are some concerns about fDOM because DOM is not the only fluorescence compound. Other pollutants that share aromaticity like polycyclic aromatic hydrocarbons and aromatic antibiotics that can produce strong fluorescence signals, which may interfere with parent source material identification.^82^

Another practical application of this analysis is in situ water quality monitoring using sensors. The improvement of field-deployable fluorometers has attracted enormous attention and has propelled catchment science by enabling scientists and managers to go beyond infrequent sampling to real-time and continuous data collection. The in situ sensors allow for a real-time view of fDOM dynamics in crucial times, such as a storm event. Sensors can record high-frequency data and ultimately give us an idea about the “first flush” of contaminants from the landscape, expressing the complex concentration–discharge hysteresis pattern.^83^ This data elucidates the sources of the pollutants and their transport pathways and measures the diurnal cycles, which are driven by biological and photochemical processes.^84^ Many sensors are currently on the market, but the current advancement only provides tools for a limited Ex/Em analysis field.^85^ This restricts research from having a comprehensive understanding of the fDOM in waterways but is a foundation for industry and agriculture to build technology specific to the parent source material for future fDOM identification. These sensors are particularly important in an agricultural context because when placed downstream of an agricultural area, they can identify the characteristics, for example, tryptophan-like fluorescence spike either from a manure spill or from a leaky slurry tank in real time.^83^ By calibrating these sensors with data from traditional water quality analyses, they can provide continuous, real-time estimates of not only DOM but also key parameters like nutrients, sediment, and even pathogens, ushering in a new era of “smart” water management.

### 5.4 Case Studies of Using fDOM Analysis in Assessing BMPs

#### 5.4.1 Case Study 1: Poultry Operations^56^

This case study reveals how fDOM analysis and PARAFAC modeling are used to evaluate advanced oxidation processes (AOPs) quantitatively as BMP in treating poultry waste. In this study, four components, such as tryptophan-like, tyrosine-like, and two humic-like fluorophores, were identified through the EEM-PARAFAC model. Among them, tryptophan-like fluorescence is a key indicator of manure-driven pollution, which is readily biodegradable. The study assessed multiple AOPs to determine their effectiveness in transforming these pollutant-associated components. Across all treatments, the results showed that tryptophan-like DOM was generally labile, which means that it degraded quickly. On the other hand, tyrosine-like DOM was more recalcitrant to most of the oxidation processes. Among all the treatments, the highest dose of hydrogen peroxide (UV–H_2_O_2_ system) was most effective in breaking down fluorescent fDOM. This study demonstrated that fDOM components and the EEM-PARAFAC characterization can serve as reliable indicators in quantifying BMP performance by tracking pollution reduction and transformation.

#### 5.4.2. Case Study 2: Organic Fertilizers^52^

In this case study, fDOM indices and components were used to quantify the influence of mineral (CaNH_4_NO_3_) and organic (pig slurry) fertilizer on the composition and amount of DOM leaching from soils and helped in evaluating the effectiveness of BMP. The quality of DOM in leachates was tracked down by BIX, FI, and the E2:E3 ratio (an indicator of molecular size). DOC concentration was reduced in both fertilization types in silt loam soil, while BIX and FI were increased, and the molecular size of DOM was decreased in the first 19 days. This result indicates enhanced microbial mineralization of soil organic matter and a reduction in DOC export. In sandy loam soil, the response was weaker, and only mineral fertilizer significantly reduced DOC and decreased the DOM molecular size. Overall, FDOM provided an easy way to quantify how fertilization BMPs affect DOM export and quality, proving that soil texture strongly controls how DOM responds to management.

## 6 Challenges and Future Perspective

There are some challenges in the practical application of fDOM sensors in the field. There are obvious differences between controlled or lab conditions and real-world situations. Relationships between fDOM and contaminants are most robust when a single, dominant source dictates the water chemistry, and the fDOM signal is chemically or physically coupled to the pollutant.^24^ A few cases where fDOM sensors could face difficulties are given as follows.

### 6.1 Interference from Suspended Particulates and Turbidity

Suspended particles, such as sediments, cause turbidity, which is a major inherent issue for fDOM sensors in field applications. For example, turbidity can cause attenuation and an underestimation of sensor readings. Suspended particles fundamentally interfere with the passage of light and detection in sensors, as the particles absorb and scatter light. This scattered light reduces the signal from both the excitation stage of the light emitter and the emission signal detected in the sensor. If this remains uncorrected, turbidity results in a dampened signal and leads to underestimating fDOM concentration.^86,87^ Turbidity is more problematic to fDOM than UV–vis sensors, and to be exact, fDOM signals decrease exponentially as turbidity increases. Saraceno et al. 2009 showed that during storm flow events at the time of peak discharge, when turbidity was highest, the unfiltered fDOM sensors underestimated the amount of fDOM by >10% than filtered sensors because of receiving dampened signals.^88^ At high turbidity levels (>1000 NTU), more than 80% of light can be attenuated in an fDOM sensor.^87^ In addition to this, the load of high suspended sediment creates practical challenges in collecting continuous fluorescence data online.^24^

### 6.2 Fluorescence Quenching (Temperature and Chemical Effects)

There are internal or external factors, listed in Table 5, for which fluorescence intensity gets reduced, and that is called quenching. Quenching makes the measurements appear artificially low.

**Table 5:** Factors Affecting fDOM Measurement

interference	mechanism and effect	sources
thermal quenching (temperature)	with the increase in temperature, electrons return to the ground state through radiationless decay and reduce the fluorescence signals. between 1 and 25 °C, fDOM values can be reduced by 0.8 to 1.5% on average with the increasing temperature per degree Celsius. this effect is critical in environments with large temperature fluctuations (e.g., > 10 °C). organic matter composition dictates the extent of thermal quenching. for example, tryptophan-like fluorophores exhibit strong thermal quenching properties	^86^ and^87^
inner filter effect (IFE)	concentrated samples (high DOC or high absorbance) absorb both the excited and emitted radiation, thus reducing emitted fluorescence intensity and distorting the band shape. high-concentration solutions need to be diluted before analysis, although it is usually negligible in natural waters (DOC < 20 mg L^–1^)	^15^,^32^, and^89^
metal quenching	metal ions (e.g., Cu^2+^, Fe^3+^, Al^3+^) form organometal complexes with fluorophores, especially humic substances that can fluorescence signature. iron is a well-known interference for DOM measurements	^32^ and^42^
ionic strength/salinity	DOM fluorescence is affected by ionic strength and salinity. studies that examined estuarine mixing mentioned that the changes in humic-like components might be a result of DOM contracting under high ionic strength conditions. riverine signals could be reduced by the salinity mixing of fresh and marine water	^37^,^42^, and^46^

### 6.3 pH Effects and Chemical Environment

Changes in pH affect fluorescence intensity over the range of pH 5–9, which is common in normal aquatic systems. Although the magnitude is fluorophore-specific, over this range, the fluorescence intensity of all fluorophores has been found to increase by about 10%.^32^ In addition to this, the peak position and the structure of the DOM are also affected by pH. In the case of humic-like substances, increasing pH from 3 to 9 was recorded to a 10 nm red shift of the peak.^90^ Soil acidity affects the chemical characteristics of DOM, and at low pH, humic substances show an increase in lipophilic nature and tendency to coagulate.^49^

### 6.4 Calibration and Methodological Difficulties

There are a few limitations that come inherently with DOM analysis related to the heterogeneity and operational nature of measurements. Proxy limitation is one of the major constraints because fDOM sensors can detect only a small fraction (approximately 1%) of the total DOM pool. That means only the fluorescent portion can be detected.^86–88^ As a result, we could have reliable information on the DOC concentration only if the measured fluorescent fraction is a dominant feature of the EEM landscape. Additionally, linear models related to the chemical parameters of the fluorescent intensity at any specific location or source are not necessarily similar for other environments. In addition, DOM that produces similar fluorescence intensities could originate from different sources and may have contrasting chemical compositions. So, fluorophores that are not universal in their chemical composition can vary greatly depending on the extent of terrestrial versus autochthonous sources.^24,45^

On the operational scale, typically, the filter pore size ranges from 0.2 to 0.7 μm, and commonly, 0.45 μm is used, which introduces inconsistencies among different studies. Besides, this filtration process could fragment the colloids as an outcome of shear forces, which might alter the measured molecular composition.^14^

Sensors and data-related artifacts could make high-frequency fDOM monitoring vulnerable. For example, the optical surface could be blocked by biofilm accumulation, and this could interfere with fluorescence measurements, requiring frequent cleaning and maintenance.^83,89,87^ Instrument-specific calibration and bias correction are essential to having the correct measurement, and this also adds further complications in the field application. In fact, often, it is recommended to use the actual water matrix, as calibrating instruments with standard solutions all the time might be operationally impractical.^71,89^ These combined limitations underscore the need for careful methodological consideration and site-specific calibration when interpreting fDOM data in natural systems.

### 6.5 Solutions and Future Research Perspectives

It is essential to have a technical solution, especially for turbidity and temperature, to reduce the major interferences that affect the fDOM measurement. To reduce particle interference, the turbidity effect can be minimized by introducing a filtration system (closed-path sensors).^35,88^ Modern programmable controllers are suggested by various studies to extend filter life, as filters might clog during long-term deployments.^86,88^ Alternatively, simultaneous deployment of an in situ turbidimeter enables the application of site-specific and sensor-specific turbidity correction equations, which are necessary, since signal reduction depends on particle composition and size.^86,89^ Temperature compensation equations should be used to correct temperature-related thermal quenching, especially in systems with large temperature fluctuations, while an alternative could be using in situ spectrophotometers when temperature could be a major concern.^86,87^ Furthermore, to fight the inner filter effect, highly concentrated samples should be diluted before running the analysis.^32,91^

Strengthening the robustness, comparability, and contextual relevance of fDOM measurements must be the goal of future methodological advancement. Scientists are encouraged to integrate fluorescence with absorbance and nutrient measurement to better capture the intrinsic DOM characteristics.^15,18,83^ Optical properties should be integrated with physical, chemical, and biological properties, such as particles, pH, temperature, etc., to provide a full picture of the DOM and to avoid misinterpretation. These are emphasized as environmental normalization in recent literature.^18^ Sharing methodological knowledge and approaches to standardization of the PARAFAC model is essential, as this will increase cross-study comparability and maybe eventually enable new samples to fit into widely accepted fluorescence models.^83,89^ At the same time, there is a continued need for standardized deployment, maintenance, and data-quality protocols.

Finally, natural and controlled systems should be linked to fDOM research in the future. PARAFAC models, which can be useful for both systems, could help in understanding how DOM behaves under natural conditions and engineered systems.^46^ Validation of fluorescence quenching or metal complexation through complementary techniques such as HR-MS or size-exclusion chromatography can confirm that observed fluorescence changes represent true transformations rather than simple shifts in fluorescence intensity.^90,92^ Development in sensor technology is needed because simple fluorometers may serve as cost-effective tools for early warning systems, but more advanced sensors will have the ability to resolve overlapping fluorophores and embed mathematical algorithms that are required for complex water environments.^90,91^ Furthermore, machine learning and artificial intelligence tools should be integrated to further advance contamination detection, monitoring efficiency, and sensor accuracy, especially in heterogeneous water matrices.^90,91,93^

## Abbreviations

fDOM: fluorescent dissolved organic matter
PARAFACs: parallel factor analysis
DOM: dissolved organic matter
POM: particulate organic matter
CDOM: colored dissolved organic matter
DOC: dissolved organic carbon
SUVA: specific UV absorbance
EEMs: excitation–emission matrices
FRI: fluorescence regional integration
EEM: excitation–emission matrix
Ex/Ex: excitation and emission wavelengths
SOM: soil organic matter
BMPs: best management practices
